# Changes in the Treatment and Outcomes of Different Severities of Neonatal Hypoxic Ischemic Encephalopathy in California: A Retrospective Cohort Study

**DOI:** 10.21203/rs.3.rs-4505263/v1

**Published:** 2024-06-17

**Authors:** Carolyn Fall, Rebecca Baer, Henry Lee, Christina Chambers, Gretchen Bandoli

**Affiliations:** University of California, San Diego; University of California San Francisco; University of California San Diego; University of California San Diego; UC San Diego Health

**Keywords:** Hypoxic Ischemic Encephalopathy, Therapeutic Hypothermia

## Abstract

**Objective:**

Evaluate the changes in management and outcomes of Californian infants with hypoxic ischemic encephalopathy (HIE).

**Study Design:**

Infants with HIE were identified from a California administrative birth cohort using ICD codes and divided into two epochs, Epoch 1 (2010–2015) and Epoch 2 (2016–2019). Risk ratios (RR) for therapeutic hypothermia (TH) in each epoch and their outcomes were calculated using log-linear regression.

**Results:**

In this cohort, 4779 infants with HIE were identified. Incidence of HIE in California increased yearly from 0.5/1,000 California births to a peak of 1.5/1,000 births in 2018. The use of TH in infants with mild HIE increased in Epoch 2 compared to Epoch 1. There was no significant difference in outcomes between epochs for infants with mild HIE that received TH.

**Conclusion:**

Significantly more infants with mild HIE received TH since 2015 in California, but no difference in outcomes was found for these patients.

## Introduction

Hypoxic ischemic encephalopathy (HIE) is a leading cause of morbidity and mortality in neonates with a previously reported incidence of 1–8/1000 births in developed countries^1^. HIE occurs when a hypoxic ischemic perinatal event such as a cord prolapse or shoulder dystocia disrupts oxygen and glucose delivery to the brain which can lead to death or severe neurological disability.^2^ Neonatal HIE is divided by severity into mild, moderate, and severe, with moderate and severe categories reported to have increased risk of mortality and severe disability such as intellectual disability and epilepsy compared to mild HIE.^3,4^

In 2005, Shankaren et al reported decreased mortality and morbidity in neonates with HIE who underwent whole body therapeutic hypothermia.^5^ Further studies followed which continued to support the safe and effective use of therapeutic hypothermia in neonates with HIE, however given that the safety of therapeutic hypothermia in neonates was not well established, these studies only included infants with moderate or severe HIE and with a gestational age greater than or equal to 36 weeks.^6,7,8,9^ Therapeutic hypothermia became the standard of care and is currently the only standard adjunctive treatment for neonatal HIE.^10^ However, by 2015 reports emerged of some hospitals cooling outside of these narrow guidelines, including infants with mild HIE despite limited evidence of effectiveness and safety.^11^ Additionally, further analysis has shown that the adverse neurological outcomes of mild HIE may be more significant than previously thought, with 16–25% of infants diagnosed with mild HIE presenting with neurological abnormalities.^12, 13^

As the second decade of the era of therapeutic hypothermia begins, there remains a gap in our knowledge in whether therapeutic hypothermia is safe and beneficial when applied to a wider patient population than was included in the initial clinical trials. While randomized control studies may be helpful in the future, a large retrospective cohort study is a readily accessible, population-based method to evaluate the changes in treatment over time of different severities of HIE and if that change in treatment has impacted outcomes of the affected infants. Previously, Bandoli et al (2022) used the same dataset as this study to evaluate maternal factors that may impact an infant receiving a diagnosis of neonatal encephalopathy and found that “substance-related diagnosis, preexisting diabetes, preeclampsia, and any maternal infection were associated with a two-fold increase in risk”.^14^ This study focuses on the postnatal treatment and outcomes of infants diagnosed with HIE to investigate the change in management of infants with different severities of HIE and their outcomes over time.

## Methods

The sample was drawn from all California live born infants between 2010 and 2019. Birth certificates, maintained by California Vital Statistics, were linked to a hospital discharge, emergency department (ED), and ambulatory surgery (AS) records maintained by the California Department of Health Care Access and Information (HCAI). Hospital discharge, ED, and AS files provide diagnoses and procedure codes based on the International Classification of Diseases, as reported to HCAI by the health care facilities. HCAI records were linked for up to one year after birth. The study sample was restricted to singleton infants born between 35- and 44-weeks gestation, with linked birth records for birthing person (“mothers”) and infant, without major anomalies, and an ICD diagnosis of neonatal HIE (Supplemental Materials). Anomalies were considered “major” if determined by clinical review as causing major morbidity and mortality that would likely be identified in the hospital at birth or lead to hospitalization during the first year of life (PMID: 24949541).

Maternal and infant characteristics obtained from birth certificates included race/ethnicity and expected payer for delivery (Medi-Cal = California’s Medicaid, health insurance for low-income persons). ‘Other race or ethnicity’ included American Indian/Alaska Native, Native Hawaiian/Pacific Islander, other race, two or more races, and not stated/unknown. Best obstetric estimate of gestational age at birth was obtained from birth certificate records and late preterm birth was defined as birth between 35 – < 37 weeks gestation. County of maternal residence, also obtained from birth certificate records, was coded as rural where the National Center for Health Statistics Urban-Rural Classification Scheme for Counties indicated the county was small metro, micropolitan, or non-core (https://www.cdc.gov/nchs/data/series/sr_02/sr02_166.pdf). Urban counties were defined as: large central metro, large fringe metro, and medium metro counties. Small for gestational age was defined as < 10th percentile for sex and gestational age and large for gestational age was defined as > 90th percentile (appropriate for gestational age was 10th to 90th percentile) (PMID: 24777216).

Infant death and age at death were obtained from linked infant death records from California Vital Statistics and/or when infant discharge status indicated ‘died’ on HCAI records. Therapeutic hypothermia, gastrostomy, tracheostomy, newborn feeding problem, subcutaneous fat necrosis/cold injury, severity of neonatal HIE, seizure/convulsions of newborn, and infant sepsis were obtained from hospital discharge/ED/AS diagnostic codes (Supplemental Materials). Infant length of stay on birth admission, transfer from birth hospital, number of readmissions, and timing of readmission were gathered and calculated from HCAI patient discharge files. Adverse outcome among infants with neonatal HIE was defined as infant birth admission stay of seven days or more, any hospital readmission, newborn feeding problem, gastrostomy, tracheostomy, subcutaneous fat necrosis/cold injury, or death within one year. Newborn feeding problem and gastrostomy placement were included to address the spectrum of severity of feeding problems affecting infants with HIE.

To examine trends, neonatal HIE rates were examined by birth year for HIE overall, by therapeutic hypothermia treatment, and by severity of HIE (mild or moderate/severe).

The sample was divided into two epochs: Epoch 1 (2010–2015) and Epoch 2 (2016–2019). The cut-point for Epoch 2 was set at 2015 as this was when initial reports of therapeutic expansion of therapeutic hypothermia in HIE began to be published. To examine trends over time, for all analyses, Epoch 2 was compared to Epoch 1.

The risk ratios (RR) and 95% confidence intervals (CI) for therapeutic hypothermia treatment compared between epochs were modeled using log-link binary regression with robust standard errors. Models were constructed for all HIE, and then stratified by severity of HIE. Adjusted risk ratios (aRR) accounted for late preterm birth, transfer from birth hospital, rural county of residence. The “all HIE” model, which includes mild, moderate/severe, and undefined/unknown severity, was further adjusted for severity of HIE.

Using the same methods, the risk ratios of selected neonatal descriptive covariates were then compared between epochs, stratifying for therapeutic hypothermia treatment. Descriptive covariates included size for gestational age (small for gestational age [SGA] or large for gestational age [LGA] versus appropriate for gestational age [AGA]), infant of diabetic mother (IDM) (yes versus no), late preterm birth (34–<37 weeks) (yes versus no), payer for delivery (private versus Medi-Cal), transfer from birth hospital (yes versus no), race or ethnicity (Hispanic, Black, Asian, or Other versus white, non-Hispanic), and seizure/convulsions of a newborn (yes versus no). Risks were estimated for all HIE, and then stratified by severity of HIE. Models were adjusted for circumstances which could impact the choice to initiate therapeutic hypothermia and included late preterm birth, transfer from birth hospital, rural county of residence. The “all HIE” model was further adjusted for severity of HIE.

Last, the risk ratios for adverse outcomes among all infants with HIE were compared between epochs by any therapeutic hypothermia treatment and then stratified by severity of HIE. Risks were adjusted for late preterm birth, transfer from birth hospital, rural county of residence. The “all HIE” model was further adjusted for severity of HIE.

All analyses were performed using Statistical Analysis Software version 9.4 (Cary, NC). Methods and protocols for the study were approved by the Committee for the Protection of Human Subjects within the Health and Human Services Agency of the State of California and by the Institutional Review Board at the University of California San Diego, and the study was performed in accordance with the Declaration of Helsinki.

## Results

### Sample Description

The sample included 4,779 infants with HIE born between 2010 and 2019. The incidence of HIE in California increased yearly from 0.5/1,000 California births in 2010 to a peak incidence of 1.5/1,000 births in 2018, followed by a slight reduction to 1.4/1,000 births in 2019. The prevalence of therapeutic hypothermia treatment among infants with HIE ranged from 19.3% in 2010 to 31.7% in 2018. Mild HIE had lower therapeutic hypothermia treatment prevalence ranging from 13.4% in 2011 to 31.3% in 2016. Prevalence of therapeutic hypothermia treatment for moderate/severe HIE ranged from 20.0% in 2010 to 40.7% in 2019 (Supplemental Table A).

The sample was approximately evenly divided between the two epochs: n = 2,310 for Epoch 1 and n = 2,469 for Epoch 2. In Epoch 1, the All HIE cohort included 12.6% mild HIE, 29.5% moderate/severe HIE, and 57.8% undefined/unknown severity of HIE. In Epoch 2, the All HIE cohort included 16.7% mild HIE, 46.6% moderate/severe, and 37.6% undefined/unknown severity of HIE. Infants with mild and moderate/severe in Epoch 2 were more likely to receive therapeutic hypothermia treatment compared to Epoch 1: 26.6% versus 18.8% for mild HIE (aRR 1.4, 95% CI 1.0 to 2.0) and 38.3%versus 30.8% for moderate/severe HIE (aRR 1.2, 95% CI 1.1, 1.5) (Table 1).

When examining neonatal descriptive covariates by epoch and stratifying by therapeutic hypothermia treatment, some differences were found. Of note, treated infants in Epoch 2 were less likely to be LGA (aRR 0.7, 95% CI 0.6 to 0.96) than treated infants in Epoch 1. Additionally, Infants who did not receive therapeutic hypothermia treatment in Epoch 2 were more likely to be IDM (aRR 1.4, 95% CI 1.1 to 1.6) or be ‘other race/ethnicity’ (aRR 1.4, 95% CI 1.1 to 1.9) and less likely to have seizure/convulsions of the newborn (aRR 0.8, 95% CI 0.7 to 0.9) (Supplemental Table B) than infants who did not receive therapeutic hypothermia in Epoch 1.

When stratifying by severity, no differences in neonatal descriptive covariates were identified between epochs among infants who received therapeutic hypothermia treatment. However, there were some differences in descriptive covariates for infants who did not receive therapeutic hypothermic when stratified by HIE severity. Infants with mild HIE in Epoch 2 who did not receive therapeutic hypothermia treatment were more likely to be IDM (aRR 1.8, 95% CI 1.1 to 2.8) (Supplemental Table C) than infants with mild HIE in Epoch 1 who did not receive therapeutic hypothermia. Infants with moderate/severe HIE in Epoch 2 who received therapeutic hypothermia treatment were less likely to have seizure/convulsions of the newborn (aRR 0.7, 95% CI 0.6 to 0.9) than infants with moderate/severe HIE in Epoch 1 who received therapeutic hypothermia. Infants with moderate/severe HIE in Epoch 2 who did not receive therapeutic hypothermia treatment were more likely to be IDM (aRR 1.4, 95% CI 1.01 to 1.9), less likely to live in a rural county (aRR 0.5, 95% CI 0.3, 0.8), and less likely to have seizure/convulsions of the newborn (aRR 0.7, 95% CI 0.6 to 0.9) (Supplemental Table D) than infants with moderate/severe HIE in Epoch 1 who did not receive therapeutic hypothermia.

### Clinical Outcomes of Infants with HIE

There were significant differences in the outcomes of infants in the “All HIE” cohort in Epoch 2 compared to infants in Epoch 1. Infants with any severity of HIE who received therapeutic hypothermia in Epoch 2 were less likely to have a long birth admission length of stay (aRR 0.8, 95% CI 0.7 to 0.9), have a hospital readmission (aRR 0.8, 95% CI 0.6, 0.98), or die within one year of birth (aRR 0.5, 95% CI 0.3 to 0.8) compared to infants with any HIE in Epoch 1 who underwent therapeutic hypothermia. Infants with any severity of HIE in Epoch 2 without therapeutic hypothermia treatment were less likely to have a long birth admission length of stay (aRR 0.8, 95% CI 0.7 to 0.9), have a hospital readmission after a week after discharge from birth admission (aRRs 0.4–0.5), have a gastrostomy (aRR 0.5, 95% CI 0.4 to 0.7), or have a tracheostomy (aRR 0.5, 95% CI 0.2 to 0.9) compared to infants with any HIE severity in Epoch 1 who did not receive therapeutic hypothermia. However, infants with any HIE severity in Epoch 2 without therapeutic hypothermia treatment were more likely to have a readmission < 4 days from birth admission discharge (aRR 1.5, 95% CI 1.3 to 1.8) and have a newborn feeding problem (aRR 1.3, 95% CI 1.1 to 1.5) (Table 2) compared to infants with any HIE in Epoch 1 who did not undergo therapeutic hypothermia.

Notably, among infants with mild HIE in Epoch 2 with therapeutic hypothermia treatment, no statistically significant differences were seen for adverse clinical outcomes when compared with infants with mild HIE in Epoch 1 who received therapeutic hypothermia, although sample sizes tended to be small. Similarly for infants with mild HIE in Epoch 2 without therapeutic hypothermia treatment, many outcomes could not be examined due to small sample sizes and the only statistically significant difference was a reduced risk of hospital readmission after 30 days (aRR 0.3, 95% CI 0.1 to 0.9) (Table 3) compared to infants with mild HIE in Epoch 1 who did not receive therapeutic hypothermia.

There were also differences in the clinical outcomes of infants with moderate/severe HIE in Epoch 2 compared to infants in Epoch 1. Infants with moderate/severe HIE in Epoch 2 who received therapeutic hypothermia treatment were less likely to have a hospital readmission (aRR 0.6, 95% CI 0.5, 0.9), or have a readmission more than 30 days after birth admission discharge (aRR 0.2, 95% CI 0.1 to 0.6) compared to infants moderate/severe HIE in Epoch 1 who underwent therapeutic hypothermia. Infants with moderate/severe HIE in Epoch 2 without therapeutic hypothermia treatment were less likely to have a hospital readmission after two weeks after discharge from birth admission (aRRs 0.2–0.4), have a gastrostomy (aRR 0.4, 95% CI 0.2 to 0.7), or die within one month of birth (aRR 0.7, 95% CI 0.5 to 0.9) compared to infants with moderate/severe HIE in Epoch 1 who did not receive therapeutic hypothermia. However, infants with moderate/severe HIE in Epoch 2 without therapeutic hypothermia treatment were more likely to have a readmission < 4 days from birth admission discharge (aRR 1.5, 95% CI 1.1 to 2.0) (Table 4) compared to those infants with moderate/severe HIE who did not receive therapeutic hypothermia in Epoch 1.

## Discussion

Since the use of therapeutic hypothermia has become standard of care for the treatment of qualifying infants with neonatal HIE, there has been controversy over whether the treatment should be offered to a wider spectrum of patients. From 2010–2019, in this state-wide cohort there was a non-monotonic increase in the incidence of infants diagnosed with HIE in California. This may indicate that the diagnosis is increasingly recognized by healthcare professionals and follows with other studies which have noted that the incidence of HIE has not decreased despite advances in the field of obstetrics and fetal monitoring.^2^ Additionally, there was increased classification of HIE as mild, moderate, or severe as opposed to undefined/unknown in Epoch 2 compared to Epoch 1, which may be related to changes in ICD coding that encourage healthcare providers to further classify HIE by severity. This study has shown that there has been a significant increase in the use of therapeutic hypothermia in infants with mild HIE in Epoch 2 compared to Epoch 1, but found no significant difference in the clinical outcomes of these infants between time periods.

Additionally, infants with moderate/severe HIE were more likely to receive therapeutic hypothermia in Epoch 2 compared to Epoch 1. While infants with any type of HIE were more likely to receive therapeutic hypothermia in the later time period, this did not reach statistical significance after adjustment. Infants with any HIE had decreased risk of certain adverse outcomes including mortality and seizures in Epoch 2 compared to Epoch 1 whether they received therapeutic hypothermia or not (Supplemental Tables B and D). This may indicate that more infants with less severe disease were being treated with therapeutic hypothermia and that advances in supportive management have improved in recent years.

While this retrospective study cannot establish why more infants with mild HIE are being cooled, we can suppose that over time there has been increased confidence with the safety profile benefit of therapeutic hypothermia which has made healthcare providers and institutions more comfortable applying it to infants that would not initially have qualified for treatment. Mild HIE itself does not have one agreed upon definition, and HIE can have a dynamic presentation with the degree of encephalopathy changing in the first few hours and days of treatment, but the decision on whether to treat or not needs to be made rapidly to have the best therapeutic effect.^15^ Others have suggested that the therapeutic creep towards the treatment of more infants with HIE is related to the fear of litigation.^16^ Given there are no standard biomarkers, imaging, or clinical markers that can reliably predict which infants HIE will develop disabilities, healthcare providers may feel they need to provide therapeutic hypothermia even to infants that do not strictly qualify for treatment in case they later develop disabilities that become a part of a lawsuit. Regardless of the reasoning behind the increased use of therapeutic hypothermia, there remains a dearth of research on the safety and effectiveness of the cooling of infants with mild HIE. Some animal studies have suggested that mild HIE may be more likely to respond to therapeutic hypothermia, while others have cautioned that animal studies have shown increased neuronal apoptosis in therapeutic hypothermia that was applied to uninjured brains.^15,16^ There may be a belief that if a treatment is beneficial, then a larger dose or longer treatment period will provide more benefit. However, studies have shown that therapeutic hypothermia does not appear to improve outcomes in a dose dependent manner. In a randomized control trial, infants with HIE cooled to a lower temperature than the standard 33.5–34.5°C were more likely to require extracorporeal membrane oxygenation and nitric oxide and infants cooled for longer than the standard 72 hours were more likely to have arrhythmias, anuria, and prolonged hospitalizations, and so the study authors discouraged cooling outside of the established neonatal HIE therapeutic hypothermia guidelines.^17^

Multiple randomized controlled trials are currently underway to further assess the effectiveness and safety of cooling in mild HIE. Ethical concerns have been raised whether there is equipoise in withholding therapeutic hypothermia for infants with mild HIE when some centers have begun to routinely treat infants with mild HIE. This study is significant for showing that the overall increase in the cooling of infants with mild HIE has not significantly impacted outcomes in the first year of life, which suggests that it is ethical to randomly assign infants with mild HIE to hypothermia or non-hypothermia groups.

This study has significant strengths due to the large population-based dataset that this cohort is derived from. This study draws from ten years of birth data covering a critical period in the change in the treatment of HIE. This long study period allows this study to clearly show trends in treatment from the beginning of the era of therapeutic hypothermia to more recent times. Another major benefit of using state derived, population-based data is that all infants in the state are included in the cohort, providing a larger denominator. Other datasets such as the California Perinatal Quality Care Collaborative (CPQCC) which also track and report outcomes of infants with HIE, are more limited as they only receive data from infants born in hospitals that participate in their data collection. However, the state-based data used in this study may also lead to over-reporting of HIE, which is likely the reason for the somewhat lower rates of therapeutic hypothermia seen in this study.

This study also had several limitations. It was a retrospective cohort study and was limited by the available records, documentation, and the use of ICD-9 and 10 codes for HIE and other diagnoses and procedures. Incomplete or inaccurate medical provider documentation of diagnoses or procedures could impact the accuracy of this study. Patients diagnosed exclusively with neonatal encephalopathy who did not also receive a code for HIE were excluded from the study due to the lack of specificity of diagnosis. The study was limited by a one-year follow-up period. Some adverse outcomes of HIE may not be diagnosed and detected by one year of life, and a longer follow-up period may demonstrate additional increased risk in this population. This study was also limited by covering a large number of hospitals over multiple years and did not determine the therapeutic hypothermia capabilities and protocols at each hospital at the time of each infant’s birth or if infants qualified for therapeutic hypothermia at the time of birth based on their birth hospital protocol and capabilities. Finally, many infants were not diagnosed specifically as mild, moderate, or severe HIE by ICD coding which limited the sample size for evaluating the effects of therapeutic hypothermia on different severities of HIE. More precise and consistent guidelines for the classification of the severity of HIE will be crucial for future studies of mild HIE.

This study demonstrated an overall increase in recognition of neonatal HIE and increased treatment of mild HIE in the state of California. This recognizes that despite the lack of a consensus national guideline on the treatment of mild HIE, more hospitals are routinely providing therapeutic hypothermia to infants with HIE in general and mild HIE in particular. However, the lack of demonstrable differences in the outcomes of these infants with mild HIE indicate that further study and randomized control trials are necessary and ethical. These studies are currently ongoing and will be beneficial in providing more substantial evidence for the treatment of mild HIE. Future directions for this dataset may include evaluating differences in the treatment and outcomes of infants with HIE in rural versus urban hospitals and community versus academic hospitals to better elucidate the environment in which the increased use of therapeutic hypothermia is occurring.

## Conclusion

This study found that significantly more infants with mild HIE received therapeutic hypothermia since 2015 compared to the period from 2010–2015. There were no significant differences in adverse outcomes for those with mild HIE who received therapeutic hypothermia over time. For infants with any HIE, there was improvement in mortality, which most likely represents improvement in technology and management over time. In the future, randomized control trials and clearer guidelines for the categorization of HIE severity would be beneficial in determining benefits of more liberal use of therapeutic hypothermia in neonatal HIE.

## Figures and Tables

**Table 1:** incidence HIE with and without TH over time

Year	With therapeutic hypothermia	Without therapeutic hypothermia		
	N (row %)	N (row %)	RR (CI)	ARR (CI)
All HIE				
Epoch 1 (2010–2015) (n = 2,310)	593 (25.7)	1,717 (74.3)	Reference	Reference
Epoch 2 (2016–2019) (n = 2,469)	745 (30.2)	1,724 (69.8)	1.18 (1.1, 1.3)	1.10 (1.0, 1.2)
Mild HIE				
Epoch 1 (2010–2015) (n = 292)	55 (18.8)	237 (81.2)	Reference	Reference
Epoch 2 (2016–2019) (n = 413)	110 (26.6)	303 (73.4)	1.41 (1.0, 2.0)	1.41 (1.0, 2.0)
Moderate/severe HIE				
Epoch 1 (2010–2015) (n = 683)	210 (30.8)	473 (69.3)	Reference	Reference
Epoch 2 (2016–2019) (n = 1,126)	431 (38.3)	695 (61.7)	1.24 (1.1, 1.5)	1.24 (1.1, 1.5)

HIE = Hypoxic Ischemia Encephalopathy, All HIE includes mild/moderate/severe and undefined/unknown severity

Adjusted for late preterm, transferred, rural, and for all HIE group, HIE severity

**Table 2:** Neonatal outcomes All HIE with and without TH over time

	With therapeutic hypothermia				Without therapeutic hypothermia			
	Epoch 1 (2010–2015)	Epoch 2 (2016–2019)			Epoch 1 (2010–2015)	Epoch 2 (2016–2019)		
	N (%)		RR (CI)	ARR (CI)	N (%)		RR (CI)	ARR (CI)
Sample	593	745			1,717	1,724		
**Any Adverse Outcome**								
No	43 (7.3)	104 (14.0)	Reference	Reference	216 (12.6)	323 (18.7)	Reference	Reference
Yes	550 (92.8)	641 (85.0)	0.9 (0.8, 1.04)	0.9 (0.8, 1.01)	1,501 (87.4)	1,401 (81.3)	**0.9 (0.8, 1.0)**	**0.9 (0.8, 1.0)**
**Length of stay for birth admission (including time at hospital of transfer)**								
< 7 days	142 (24.0)	265 (35.6)	Reference	Reference	598 (34.8)	760 (44.1)	Reference	Reference
Any long stay	392 (66.1)	400 (53.7)	**0.8 (0.7, 0.9)**	**0.8 (0.7, 0.9)**	882 (51.4)	725 (42.1)	**0.8 (0.7, 0.9)**	**0.8 (0.7, 0.9)**
7 – < 14 days	229 (38.6)	289 (38.8)	0.9 (0.7, 1.05)	0.9 (0.7, 1.08)	477 (27.8)	483 (28.0)	0.9 (0.8, 1.0)	0.9 (0.8, 1.0)
14 – < 30 days	134 (22.6)	145 (19.5)	**0.7 (0.6, 0.9)**	**0.6 (0.5, 0.8)**	330 (19.2)	273 (15.8)	**0.8 (0.6, 0.9)**	**0.7 (0.6, 0.8)**
30 + days	54 (9.1)	40 (5.4)	**0.5 (0.3, 0.7)**	**0.4 (0.2, 0.6)**	132 (7.7)	105 (6.1)	**0.7 (0.5, 0.9)**	**0.5 (0.4, 0.7)**
**Number of readmissions in first year of life**								
None	416 (70.2)	539 (72.4)	Reference	Reference	1,147 (66.8)	1,143 (66.3)	Reference	Reference
Any	177 (29.9)	206 (27.7)	0.9 (0.8, 1.1)	**0.8 (0.6, 1.0)**	570 (33.2)	581 (33.7)	1.0 (0.9, 1.1)	0.9 (0.8, 1.1)
1	137 (23.1)	144 (19.3)	0.9 (0.7, 1.1)	**0.7 (0.5, 0.9)**	408 (23.8)	388 (22.5)	1.0 (0.8, 1.1)	0.9 (0.8, 1.04)
2	27 (4.6)	34 (4.6)	1.0 (0.6, 1.6)	0.8 (0.5, 1.5)	91 (5.3)	105 (6.1)	1.1 (0.9, 1.5)	1.1 (0.8, 1.4)
3+	13 (2.2)	28 (3.8)	1.6 (0.8, 3.1)	1.2 (0.6, 2.4)	71 (4.1)	88 (5.1)	1.2 (0.9, 1.7)	1.1 (0.8, 1.5)
**Time to first readmission after discharge from birth admission**								
No readmissions	416 (70.2)	539 (72.4)	Reference	Reference	1,147 (66.8)	1,143 (66.3)	Reference	Reference
< 4 days	120 (20.2)	169 (22.7)	1.1 (0.8, 1.3)	0.9 (0.7, 1.1)	234 (13.6)	447 (25.9)	**1.7 (1.4, 1.9)**	**1.5 (1.3, 1.8)**
4 – < 7 days	12 (2.0)	*	**0.3 (0.1, 0.9)**	0.5 (0.2, 1.4)	20 (1.2)	15 (0.9)	0.8 (0.5, 1.5)	0.7 (0.3, 1.5)
7 –- < 14 days	15 (2.5)	*	**0.4 (0.2, 0.9)**	**0.3 (0.1, 0.8)**	44 (2.6)	20 (1.2)	**0.5 (0.3, 0.8)**	**0.5 (0.3, 0.8)**
14 – < 30 days	*	*	0.8 (0.3, 1.9)	0.4 (0.1, 1.1)	55 (3.2)	25 (1.5)	**0.5 (0.3, 0.8)**	**0.4 (0.2, 0.7)**
30 + days	20 (3.4)	15 (2.0)	0.6 (0.3, 1.2)	0.5 (0.2, 1.1)	217 (12.6)	74 (4.3)	**0.4 (0.3, 0.5)**	**0.4 (0.3, 0.5)**
**Gastrostomy**								
No	552 (93.1)	745 (100.0)	Reference	Reference	1,623 (94.5)	1,724 (100.0)	Reference	Reference
Yes	41 (6.9)	*	n/c	n/c	104 (6.1)	57 (3.3)	**0.5 (0.4, 0.8)**	**0.5 (0.4, 0.7)**
**Tracheostomy**								
No	591 (99.7)	745 (100.0)	Reference	Reference	1,699 (99.9)	1,724 (100.0)	Reference	Reference
Yes	*	*	n/c	n/c	31 (1.8)	16 (0.9)	**0.5 (0.3, 0.9)**	**0.5 (0.2, 0.9)**
**SC Fat Necrosis/Cold Injury**								
No	585 (98.7)	729 (97.9)	Reference	Reference	1,704 (99.2)	1,709 (99.1)	Reference	Reference
Yes	*	16 (2.2)	1.5 (0.7, 3.7)	1.2 (0.5, 3.3)	13 (0.8)	15 (0.9)	1.1 (0.5, 2.4)	1.0 (0.4, 2.3)
**Newborn feeding problem**								
No	410 (69.1)	444 (59.6)	Reference	Reference	1,324 (77.1)	1,188 (68.9)	Reference	Reference
Yes	183 (30.9)	301 (40.4)	**1.3 (1.1, 1.6)**	1.1 (0.9, 1.4)	393 (22.9)	536 (31.1)	**1.4 (1.2, 1.5)**	**1.3 (1.1, 1.5)**
**Mortality**								
1 Month	46 (7.8)	37 (5.0)	**0.6 (0.4, 1.0)**	**0.5 (0.3, 0.8)**	172 (10.0)	145 (8.4)	0.8 (0.7, 1.1)	**0.7 (0.5, 0.9)**
1 Year	53 (8.9)	42 (5.6)	**0.6 (0.4, 0.9)**	**0.5 (0.3, 0.8)**	216 (12.6)	199 (11.5)	0.9 (0.8, 1.1)	**0.8 (0.6, 1.0)**

HIE = Hypoxic Ischemia Encephalopathy, TH = Therapeutic Hypothermia, SC = Sub-cutaneous,

* not displayed when n < 11, All HIE includes mild/moderate/severe and undefined/unknown severity

**Table 3:** Neonatal outcomes Mild HIE with and without TH over time

	With therapeutic hypothermia				Without therapeutic hypothermia			
	Epoch 1 (2010–2015)	Epoch 2 (2016–2019)			Epoch 1 (2010–2015)	Epoch 2 (2016–2019)		
	N (%)		RR (CI)	ARR (CI)	N (%)		RR (CI)	ARR (CI)
Sample	55	110			237	303		
**Any Adverse Outcome**								
No	*	22 (20.0)	Reference	Reference	59 (24.9)	86 (28.4)	Reference	Reference
Yes	50 (90.9)	88 (80.0)	0.9 (0.6, 1.2)	0.8 (0.6, 1.2)	178 (75.1)	217 (71.6)	1.0 (0.8, 1.2)	0.9 (0.7, 1.1)
**Length of stay for birth admission (including time at hospital of transfer)**								
< 7 days	11 (20.0)	45 (40.9)	Reference	Reference	113 (47.7)	158 (52.2)	Reference	Reference
Any long stay	41 (74.6)	56 (50.9)	0.7 (0.5, 1.1)	0.7 (0.4, 1.04)	113 (47.7)	128 (42.2)	0.9 (0.7, 1.2)	0.8 (0.6, 1.1)
7 – < 14 days	29 (52.7)	43 (39.10	0.7 (0.4, 1.1)	0.7 (0.3, 1.6)	77 (32.5)	94 (31.0)	0.9 (0.7, 1.3)	0.9 (0.6, 1.2)
14 – < 30 days	*	22 (20.0)	0.7 (0.3, 1.5)	0.7 (0.3, 1.5)	34 (14.4)	40 (13.2)	0.9 (0.6, 1.4)	0.7 (0.4, 1.2)
30 + days	*	*	n/c	n/c	*	11 (3.6)	0.9 (0.4, 2.1)	0.7 (0.3, 1.8)
**Number of readmissions in first year of life**								
None	41 (74.6)	84 (76.4)	Reference	Reference	176 (74.3)	223 (73.6)	Reference	Reference
Any	14 (25.5)	26 (23.6)	0.9 (0.5, 1.8)	0.7 (0.4, 1.5)	61 (25.7)	80 (26.4)	1.0 (0.7, 1.4)	0.9 (0.6, 1.2)
1	*	19 (17.3)	0.9 (0.4, 2.0)	0.8 (0.3, 1.8)	53 (22.4)	56 (18.5)	0.9 (0.6, 1.3)	0.7 (0.5, 1.1)
2	*	*	n/c	n/c	*	13 (4.3)	1.4 (0.6, 3.6)	1.2 (0.5, 3.3)
3+	*	*	n/c	n/c	*	11 (3.6)	n/c	n/c
**Time to first readmission after discharge from birth admission**								
No readmissions	41 (74.6)	84 (76.4)	Reference	Reference	176 (74.3)	223 (73.6)	Reference	Reference
< 4 days	12 (21.8)	22 (20.0)	1.0 (0.2, 5.3)	0.7 (0.3, 1.5)	31 (13.1)	63 (20.8)	1.5 (0.96, 2.3)	1.2 (0.7, 1.9)
4 – < 7 days	*	*	n/c	n/c	*	*	n/c	n/c
7 – < 14 days	*	*	n/c	n/c	*	*	n/c	n/c
14 – < 30 days	*	*	n/c	n/c	*	8	n/c	n/c
30 + days	*	*	n/c	n/c	15 (6.3)	*	**0.4 (0.2, 0.95)**	**0.3 (0.1, 0.9)**
**Gastrostomy**								
No	53 (96.4)	110 (100.0)	Reference	Reference	235 (99.2)	300 (99.10)	Reference	Reference
Yes	*	*	n/c	n/c	*	*	n/c	n/c
**Tracheostomy**								
No	54 (98.2)	110 (100.0)	Reference	Reference	237 (100.0)	302 (99.7)	Reference	Reference
Yes	*	*	n/c	n/c	*	*	n/c	n/c
**SC Fat Necrosis/Cold Injury**								
No	54 (98.2)	100 (100.0)	Reference	Reference	236 (99.6)	299 (98.7)	Reference	Reference
Yes	*	*	n/c	n/c	*	*	n/c	n/c
**Newborn feeding problem**								
No	38 (69.1)	70 (63.6)	Reference	Reference	189 (79.8)	208 (68.7)	Reference	Reference
Yes	17 (30.9)	40 (36.4)	1.2 (0.7, 2.1)	1.0 (0.5, 1.8)	48 (20.3)	95 (31.4)	**1.5 (1.1, 2.2)**	1.3 (0.9, 1.9)
**Mortality**								
1 Month	*	*	n/c	n/c	*	*	n/c	n/c
1 Year	*	*	n/c	n/c	*	*	n/c	n/c

HIE = Hypoxic Ischemia Encephalopathy, TH = Therapeutic Hypothermia, SC = Sub-cutaneous,

* not displayed when n < 11

**Table 4:** Neonatal outcomes Moderate/Severe HIE with and without TH over time

	With therapeutic hypothermia				Without therapeutic hypothermia			
	Epoch 1 (2010–2015)	Epoch 2 (2016–2019)			Epoch 1 (2010–2015)	Epoch 2 (2016–2019)		
	N (%)		RR (CI)	ARR (CI)	N (%)		RR (CI)	ARR (CI)
Sample	210	431			473	695		
**Any Adverse Outcome**								
No	13 (6.2)	47 (10.9)	Reference	Reference	35 (7.4)	91 (13.1)	Reference	Reference
Yes	197 (93.8)	384 (89.1)	0.9 (0.8, 1.1)	0.9 (0.8, 1.1)	438 (92.6)	604 (86.9)	0.9 (0.8, 1.1)	0.9 (0.8, 1.1)
**Length of stay for birth admission (including time at hospital of transfer)**								
< 7 days	51 (24.3)	137 (31.8)	Reference	Reference	136 (28.8)	267 (38.4)	Reference	Reference
Any long stay	130 (61.9)	247 (57.3)	0.9 (0.7, 1.1)	0.9 (0.7, 1.1)	235 (49.7)	309 (44.5)	0.8 (0.7, 1.004)	**0.8 (0.7, 1.0)**
7 – < 14 days	66 (31.4)	165 (38.3)	1.0 (0.8, 1.3)	1.0 (0.7, 1.3)	118 (25.0)	182 (26.2)	0.9 (0.7, 1.1)	0.9 (0.7, 1.1)
14 – < 30 days	57 (27.1)	95 (22.0)	0.8 (0.6, 1.1)	0.7 (0.5, 1.003)	96 (20.3)	134 (19.3)	0.8 (0.6, 1.1)	0.8 (0.6, 1.1)
30 + days	16 (7.6)	31 (7.2)	0.8 (0.4, 1.4)	0.6 90.3, 1.3)	38 (8.0)	55 (7.9)	0.8 (0.5, 1.2)	**0.6 (0.4, 1.0)**
**Number of readmissions in first year of life**								
None	135 (64.3)	314 (72.9)	Reference	Reference	309 (65.3)	454 (65.3)	Reference	Reference
Any	75 (35.7)	117 (27.2)	0.8 (0.6, 1.02)	**0.6 (0.5, 0.9)**	164 (34.7)	241 (34.7)	1.0 (0.8, 1.2)	1.0 (0.8, 1.2)
1	61 (29.1)	79 (18.3)	**0.6 (0.5, 0.9)**	**0.5 (0.3, 0.7)**	107 (22.6)	164 (23.6)	1.0 (0.8, 1.3)	1.0 (0.8, 1.3)
2	*	20 (4.6)	1.4 (0.6, 3.5)	1.4 (0.5, 3.7)	35 (7.4)	38 (5.5)	0.8 (0.5, 1.2)	0.7 (0.4, 1.1)
3+	*	18 (4.2)	1.0 (0.4, 2.2)	0.9 (0.4, 2.3)	22 (4.7)	39 (5.6)	1.2 (0.7, 2.0)	1.3 (0.8, 2.3)
**Time to first readmission after discharge from birth admission**								
No readmissions	135 (64.3)	314 (72.9)	Reference	Reference	309 (65.3)	454 (65.3)	Reference	Reference
< 4 days	45 (21.4)	99 (23.0)	1.0 (0.7, 1.4)	0.8 (0.5, 1.2)	76 (16.1)	188 (27.1)	**1.5 (1.1, 1.9)**	**1.5 (1.1, 2.0)**
4 – < 7 days	*	*	n/c	n/c	*	*	n/c	n/c
7 – < 14 days	*	*	n/c	n/c	14 (3.0)	*	0.5 (0.2, 1.1)	0.4 (0.2, 1.1)
14 – < 30 days	*	*	0.4 (0.1, 1.5)	0.3 (0.1, 1.5)	20 (4.2)	*	**0.3 (0.1, 0.6)**	**0.2 (0.1, 0.6)**
30 + days	*	*	**0.4 (0.1, 0.9)**	**0.2 (0.1, 0.6)**	51 (10.8)	30 (4.3)	**0.4 (0.3, 0.7)**	**0.4 (0.2, 0.7)**
**Gastrostomy**								
No	194 (92.4)	427 (99.1)	Reference	Reference	431 (91.1)	695 (100.0)	Reference	Reference
Yes	16 (7.6)	*	n/c	n/c	45 (9.5)	25 (3.6)	**0.4 (0.2, 0.6)**	**0.4 (0.2, 0.7)**
**Tracheostomy**								
No	209 (99.5)	431 (100.0)	Reference	Reference	465 (98.3)	695 (100.0)	Reference	Reference
Yes	*	*	n/c	n/c	14 (3.0)	*	0.4 (0.2, 1.0)	0.5 (0.2, 1.1)
**SC Fat Necrosis/ Cold Injury**								
No	209 (99.5)	421 (97.7)	Reference	Reference	470 (99.40	685 (98.6)	Reference	Reference
Yes	*	*	n/c	n/c	*	*	n/c	n/c
**Newborn feeding problem**								
No	141 (67.1)	237 (55.0)	Reference	Reference	360 (76.1)	480 (69.1)	Reference	Reference
Yes	69 (32.9)	194 (45.0)	**1.4 (1.04, 1.8)**	1.3 (0.9, 1.7)	113 (23.9)	215 (30.9)	**1.3 (1.03, 1.6)**	1.2 (0.96, 1.6)
**Mortality**								
1 Month	29 (13.8)	31 (7.2)	**0.5 (0.3, 0.9)**	**0.6 (0.3, 1.0)**	102 (21.6)	100 (14.4)	**0.7 (0.5, 0.9)**	**0.7 (0.5, 0.9)**
1 Year	32 (15.2)	35 (8.1)	**0.5 (0.3, 0.9)**	0.6 (0.4, 1.1)	117 (24.7)	130 (18.7)	**0.8 (0.6, 1.0)**	0.8 (0.6, 1.0)

HIE = Hypoxic Ischemia Encephalopathy, TH = Therapeutic Hypothermia, SC = Sub-cutaneous,

* not displayed when n < 11
